# Reduction in Lp(a) after a medically supervised, prolonged water-only fast followed by a whole-plant-food diet free of added salt, oil, and sugar: a case report

**DOI:** 10.3389/fnut.2024.1418705

**Published:** 2024-09-24

**Authors:** Natasha Thompson, Anthony Streutker, Alan C. Goldhamer, Toshia R. Myers

**Affiliations:** ^1^TrueNorth Health Foundation, Santa Rosa, CA, United States; ^2^TrueNorth Health Center, Santa Rosa, CA, United States

**Keywords:** lipoprotein(a), water-only fasting, prolonged fasting, whole-plant-food diet, case report

## Abstract

Lipoprotein(a) [Lp(a)] is a low-density lipoprotein (LDL) associated with increased cardiovascular disease (CVD) risk. High Lp(a) levels are genetically determined and lack effective pharmacotherapy. This case report describes a 67-year-old, vegan male with elevated blood pressure (BP), total cholesterol (TC), LDL, and Lp(a) who underwent a 10-day, medically supervised water-only fast followed by a 6-week SOS-free diet (free of added salt, oil, and sugar). At the 6-week-follow-up visit, he experienced significant reductions in several CVD risk markers, including blood pressure, total cholesterol, LDL, and high-sensitivity C-reactive protein. He also experienced an unexpected decrease in Lp(a), from 236.3 nmol/L to 143.4 nmol/L (39%). This decrease is comparable to reductions achieved with proprotein convertase subtilisin/kexin type 9 (PCSK9) inhibitors. These findings suggest that prolonged water-only fasting and/or an SOS-free diet may be an effective alternative approach for managing high Lp(a) levels and reducing CVD risk in a vegan population, warranting further research.

## Introduction

Lipoprotein(a) [Lp(a)] is a type of low-density lipoprotein (LDL) that has been positively correlated with cardiovascular disease (CVD) since the early 2000s (1, 2). High Lp(a) is typically defined as concentrations >50 mg/dL or > 125 nmol/L (2–5), although lifetime risk of major cardiovascular events increases linearly with increasing Lp(a) even below this threshold (6). The estimated global prevalence of high Lp(a) is ~20% (2). Recent Mendelian randomization studies suggest that individual Lp(a) concentrations are genetically determined and that high concentrations are an independent and causal risk factor for the development of a variety of CVDs (5, 7, 8). For example, high Lp(a) concentrations reportedly confer a 2–3-fold increase in myocardial infarction, peripheral artery disease, and aortic valve stenosis relative to low concentrations (2). There is currently a lack of approved pharmacotherapy and it appears that standard diet and lifestyle recommendations do not sufficiently lower high Lp(a) (2, 9). It has also not been conclusively established if lowering Lp(a) improves CVD outcomes (2, 10).

Nevertheless, the moderate prevalence and presumed causal increase in CVD associated with high Lp(a) indicate that a treatment which naturally lowers Lp(a) may be beneficial. Medically supervised, prolonged water-only fasting is an established and safe method of therapeutic fasting during which patients consume only water for up to 40 days followed by a five-phase refeeding diet of half the fast length (11). The refeeding diet consists exclusively of whole-plant foods free of added salt, oil, and sugar (SOS-free diet). Observational trials suggest that this “fasting/refeeding” intervention is associated with sustained reductions in various markers of CVD risk, namely high blood pressure and excess body weight (12, 13). However, there are no reports on how fasting/refeeding affect Lp(a).

Here we report the case of a 67-year-old male, with increased CVD risk, who completed a 10-day water-only fast followed by strict adherence to an SOS-free diet for the next 6 weeks. During that time, the patient had expected improvements in markers of CVD risk as well as an unexpected reduction in Lp(a). To our knowledge, this is the first report of prolonged water-only fasting followed by an SOS-free diet being associated with a reduction in Lp(a) and suggests that the intervention may be an alternative approach for managing high Lp(a) levels.

## Case presentation

A 67-year-old, White, non-Hispanic male arrived to the residential fasting center with concerns of high blood pressure and recent weight gain despite eating a whole-food vegan diet for the past 33 years and running 30 miles each week. He did not have any formal medical diagnoses and was not taking any medications. On arrival, he had consecutive systolic/diastolic blood pressure readings higher than 140/80 mmHg, his body mass index (BMI) was 23.62 kg/m^2^, and he complained of chronic right shoulder pain. Laboratory tests taken three-days prior to arrival indicated that his total cholesterol (TC), LDL, and Lp(a) were elevated and very-low-density lipoprotein (VLDL), high-sensitivity C-reactive protein (hsCRP), and hemoglobin A1C (HbA1C) were in the high-normal range (Table 1). He also reported a history of chronic, idiopathic elevated alkaline phosphatase and total bilirubin (Table 2). His family history included cardiovascular disease in both parents, which resulted in a myocardial infarction in his father at age 88 and a cerebrovascular accident in his mother at age 95.

**Table 1:** Changes in biomarkers after a 10-day water-only fast followed by SOS-free diet.

	Before fast	6-week follow-up	% Change
BW, kg	77.4	72.6	−6%
SBP/DBP, mmHg (<120/80 mmHg)	**145/82**	106/65	−27%/−21%
BMI, kg/m^2^ (18.5–24.9 kg/m^2^)	23.62	22.2	−6%
TC, mmol/L (2.59–5.15 mmol/L)	**6.52**	4.45	−32%
HDL, mmol/L (≥1.01 mmol/L)	1.63	1.32	−19%
TC/HDL ratio (0.0–5.0)	4	3.4	−15%
LDL, mmol/L (< 2.56 mmol/L)	**4.2**	**2.6**	−38%
VLDL, mmol/L (<0.78 mmol/L)	0.7	0.52	−26%
TGs, mmol/L (<3.86 mmol/L)	1.68	1.26	−25%
Lp(a), nmol/L (<125 nmol/L)	**236.2**	**143.4**	−39%
hsCRP, nmol/L (< 28.6 nmol/L)	**13.9**	2.95	−79%
HgbA1c, % (4.8–5.6%)	5.2	4.9	−6%

Reference intervals are below the respective variable. Numbers in bold exceed the reference range; SBP, Systolic blood pressure; DBP, Diastolic blood pressure; BW, Body weight; BMI, Body mass index; TC, Total cholesterol; HDL, High-density lipoprotein; LDL, Low-density lipoprotein; VLDL, Very-low density lipoprotein; TG, Triglycerides; Lp(a), Lipoprotein(a); hsCRP, High-sensitivity C-reactive protein; HgbA1c, Hemoglobin A1c; kg, Kilogram; m, Meter; cm, Centimeter; mmHg, Millimeters of mercury; mmol, Millimoler; L, Liter; nmol, Nanomole; and %, Percent.

**Table 2:** Complete blood count and comprehensive metabolic panel before, during, and after fasting.

	Baseline	Fasting day 5	Fasting day 8	6-week follow-up
WBC, x10E3/μL (3.4–10.8)	7.5	5.8	n/a	5.1
RBC, x10E6/μL (4.14–5.80)	5.17	5.49	n/a	4.73
Hemoglobin, g/dL (13.0–17.7)	15.8	16.7	n/a	14.9
Hematocrit, % (37.5–51.0)	47.2	48.4	n/a	44.9
MCV, fL (79–97)	91	88	n/a	95
MCH, pg. (26.6–33.0)	30.6	30.4	n/a	31.5
MCHC, g/dL (31.5–35.7)	33.5	34.5	n/a	33.2
RDW, % (11.6–15.4)	12.5	12.5	n/a	12.6
Platelets, x10E3/μL (150–450)	298	297	n/a	241
Neutrophils, % (Not established)	70	63	n/a	67
Lymphocytes, % (Not established)	20	23	n/a	22
Monocytes, % (Not established)	8	11	n/a	8
Eosinophils, % (Not established)	2	2	n/a	2
Basophils, % (Not established)	0	1	n/a	1
Neutrophils (Absolute), x10E3/μL (1.4–7.0)	5.2	3.6	n/a	3.4
Lymphocytes (Absolute), x10E3/μL (0.7–3.1)	1.5	1.4	n/a	1.1
Monocytes (Absolute), x10E3/μL (0.1–0.9)	0.6	0.6	n/a	0.4
Eosinophils (Absolute), x10E3/μL (0.0–0.4)	0.1	0.1	n/a	0.1
Basophils, (Absolute), x10E3/μL (0.0–0.2)	0	0	n/a	0
Immature granulocytes, % (Not established)	0	0	n/a	0
Immature granulocytes (Absolute), x10E3/μL (0.0–0.1)	0	0	n/a	0
Glucose, mg/dL (70–99)	81	**55**	**66**	85
BUN, mg/dL (8–27)	11	14	11	9
Creatinine, mg/dL (0.76–1.27)	0.78	0.89	1.06	0.89
eGFR, mL/min/1.73 (>59)	98	94	77	94
BUN/Creatinine ratio (10–24)	14	16	10	10
Sodium, mmol/L (134–144)	140	**133**	136	142
Potassium mmol/L (3.5–5.2)	5.1	4.3	5.1	4.8
Chloride mmol/L (96–106)	101	96	96	103
Carbon dioxide, Total, mmol/L (20–29)	26	**16**	**8**	24
Calcium, mg/dL (8.6–10.2)	9.8	10.2	**10.7**	9.8
Protein, Total, g/dL (6.0–8.5)	7.1	7.5	8	6.9
Albumin, g/dL (3.9–4.9)	4.3	4.6	4.8	4.2
Globulin, Total, g/dL (1.5–4.5)	2.8	2.9	3.2	2.7
A/G ratio (1.2–2.2)	1.5	1.6	1.5	1.6
Bilirubin, Total, mg/dL (0.0–1.2)	**1.5**	**4.4**	**2.9**	**1.9**
Alkaline phosphatase, IU/L (44–121)	**131**	**129**	**129**	102
LDH, IU/L (121–224)	166	143	n/a	181
AST, IU/L (0–40)	25	20	**42**	17
ALT, IU/L (0–44)	22	15	20	18

Reference intervals are below the respective variable. Numbers in bold exceed the reference range. WBC, White blood cells; RBC, Red blood cells; MCV, Mean corpuscular volume; MCH, Mean corpuscular hemoglobin; MCHC, Mean corpuscular hemoglobin concentration; RDW, Red blood cell distribution width; BUN, Blood urea nitrogen; eGFR, Estimated glomerular filtration rate; a/G ratio, Albumin/globulin ratio; LDH, Lactate dehydrogenase; AST, Aspartate transferase; ALT, Alanine aminotransferase; μL, Microliters; g, Grams; dL, Deciliter; %, Percent; fL, Femtoliter; pg., Pictograms; mg, Milligrams; mL, Milliliters; min, Minute; mmol, Millimole; L, Liter; IU, International units; n/a, Not available.

**Figure 1:**
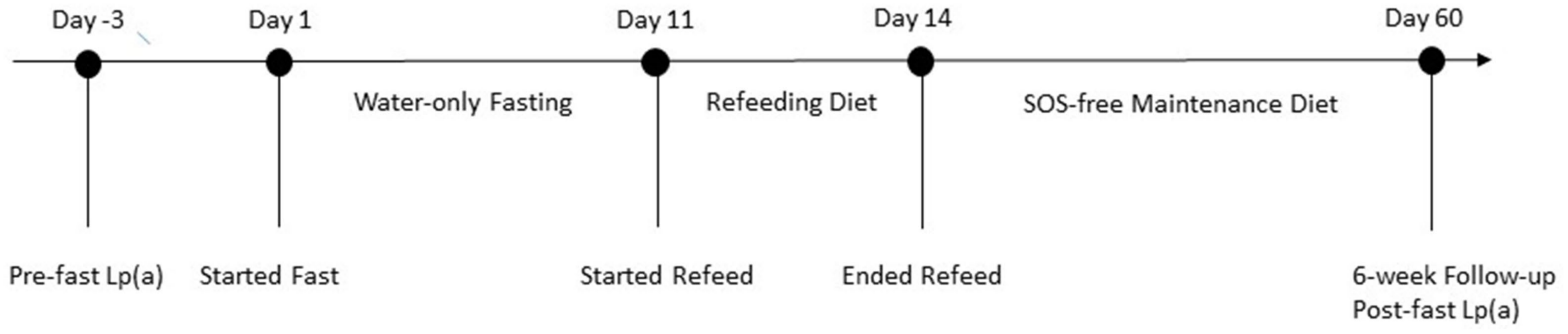
Timeline of case presentation.

In order to determine if the patient had contraindications to fasting, the attending medical doctor conducted a thorough medical examination, including complete blood count (CBC) and comprehensive metabolic panel (CMP; Table 2). The patient was approved to fast and completed a 10 days of water-only fasting. He terminated the fast with a supervised, five-phase refeeding protocol with each phase lasting 1 day for every 7 days of fasting for a total of 5 days. The first phase consisted of fresh fruit and vegetable juices, followed by the addition of raw fruits and vegetables, then the addition of steamed vegetables, then the addition of cooked gluten-free, whole-grains, and ending with an exclusively SOS-free diet. The patient completed the final day of refeeding offsite (Figure 1). While at the residential fasting center, the patient had access to 24-h medical supervision, was monitored twice daily to check vital signs and inquire on fasting-related symptoms, and completed a CBC on fasting day 5 and CMP on fasting days 5 and 8 (Table 2). As expected, the patient’s blood glucose and carbon dioxide levels dropped below normal during fasting. He also had one slightly low sodium reading of 133 mmol/L on fasting day 5, which corrected without intervention. On fasting day 8 he had slightly elevated calcium of 10.7 mg/dL and elevated aspartate transferase (AST) of 42 IU/L, which resolved by the 6-week follow-up visit (Table 2). Throughout the intervention, he had elevated total bilirubin and alkaline phosphatase, consistent with his medical history. Except for mild light-headedness upon rising, which resolved with refeeding, he did not experience any other adverse events. During the fast, the patient received chiropractic care, which reportedly improved his shoulder pain. By the end of the intervention, he had lost 6.1 kg of body weight with a BMI of 21.78 and systolic/diastolic blood pressure of 93/61 mmHg (Table 1).

After the leaving the center, the patient reported strict adherence to an SOS-free diet consisting of fruits, vegetables, legumes, grains, nuts, and seeds without added salt, oil, and sugar and necessary supplementation with vitamin B12. Six weeks post-intervention, the patient completed another CBC and CMP, which indicated that all values, except bilirubin, were within normal rage (Table 2). His chronically elevated alkaline phosphatase had also normalized. Additionally, his TC reduced to within normal range, LDL dropped 38% to 2.6 nmol/L, and Lp(a) reduced 39%, from 236.3 nmol/L to 143.4 nmol/L. His hsCRP, HbA1C, and VDL also dropped from the high-normal range to the low- to mid-normal range. He experienced a slight reduction in high-density lipoprotein (HDL) but it remained within normal range. Over the 6-week period, he regained 1.3 kg and maintained a BMI of 22.2 and systolic/diastolic BP of 106/65 mmHg (Table 1). Overall, the patient reported that the intervention was tolerable and was pleased with the results, which indicate an improvement in CVD risk.

## Discussion

The patient in this report underwent a single, 10-day water-only fast followed by strict adherence to an SOS-free diet for the next 6 weeks. During that time, he experienced sustained reductions in several CVD risk factors, including BW, BP, TC, LDL, and Lp(a). Except for Lp(a), all of these markers have been reported to improve with prolonged and intermittent fasting interventions (12, 14, 15). Indeed, BW and BP were measured daily throughout the in-patient intervention demonstrating that water-only fasting contributed to these improvements. Since the additional markers were only assessed 6 weeks after the fasting intervention ended, it is difficult to differentiate the effects that fasting and/or diet had on these outcomes. Nevertheless, clinical research, indicates that hsCRP, TC, and LDL increase during fasting but drop to below pre-fast values within days to weeks after fasting ends, which likely happened in this patient as well (12, 16).

There are currently no other reports describing the effects of fasting on Lp(a), but there is one report of a male physician who independently found that he could repeatedly lower his high Lp(a) by eating a very-low-calorie-ketogenic diet and raise it by eating a high-carbohydrate diet (17). Similar to water-only fasting, ketogenic diets significantly reduce carbohydrate intake, which subsequently increases rates of ketosis and lipolysis. Although carbohydrate intake appears to be the catalyst in that case, it is unknown if the change is a direct (e.g., decreased insulin) or indirect (e.g., increased lipolysis) result of decreased carbohydrate consumption. The effect of nutrition on Lp(a) is largely inconclusive, but limited research supports the notion that carbohydrate intake affects Lp(a) concentrations. For example, a low-fat, high-vegetable diet resulted in significant 9% increase in Lp(a) in 37 healthy women and various “Dietary Approaches to Stop Hypertension” (DASH)-type diets increased Lp(a) by 8–19% in 155 men and women (18). Whereas another study found that a eating a carbohydrate restricted diet (13% of total calories) for 12 weeks lowered Lp(a) by 11% (19). The SOS-free diet that the patient consumed after fasting is comprised of up to 70–75% carbohydrates, 10–12% protein, and 15–18% fat, and it is considered a high-carbohydrate diet. Thus, it seems likely that the 39% reduction in Lp(a) observed in this case resulted from fasting or a combination of fasting and diet rather than dietary change alone.

Lipid apheresis in the only FDA approved treatment to address high Lp(a) concentrations, and it is only approved for use in patients with CVD, familial hypercholesterolemia, and LDL > 100 mg/dL (9). Lipid apheresis has demonstrated large (~70%) reductions in Lp(a), as well as approximately 60–86% reductions in yearly major adverse cardiac events (20, 21). However, the majority (98%) of patients with high Lp(a) do not qualify for this treatment since familial hypercholesterolemia has an estimated prevalence of 1 in 250 people (22) whereas one in five people have high Lp(a) (4, 5). In addition, the procedure is costly and time-consuming, and the benefits cannot be attributed solely to Lp(a) reduction as it also removes other LDL particles and triglycerides from the blood. Pharmacologic therapies that decrease Lp(a) by 19–37% are undergoing phase 2 and phase 3 trials, but they have not consistently shown beneficial CVD outcomes (10). However, one large-scale randomized placebo-controlled trial testing evolocumab, an injectable monoclonal antibody that inhibits PCSK9 and reduces Lp(a) by 16–33%, demonstrated a 23% relative reduction in coronary heart disease death, myocardial infarction, or urgent coronary revascularization in patients with established CVD and baseline Lp(a) concentrations >120 nmol/L (23). Additionally, genetic epidemiologic modeling studies estimate that lowering Lp(a) by 105–215 nmol/L is necessary to achieve clinical benefit (24–26). This patient achieved a 93 nmol/L (39%) reduction with a single fasting/refeeding intervention, which may result in clinically significant outcomes and warrants further inquiry.

While the results are encouraging, this report has several limitations. Firstly, Lp(a) levels were not measured during or immediately after fasting, making it difficult to determine whether the reduction in Lp(a) was due to fasting, adherence to an SOS-free diet, or a combination of both. Future studies should measure Lp(a) at additional time points, including at the end of fasting, and use tools to accurately track dietary intake. Secondly, the long-term effects of an SOS-free diet are not well documented and may contradict current research (27–29). Follow-up longer than 6 weeks is necessary to determine the durability of outcomes and monitor for new or sustained adverse events including potential vitamin and mineral deficiencies. Lastly, this report does not establish causality or determine if lowering Lp(a) through this intervention reduces the risk of developing CVD. Further research to explore these aspects and confirm the findings is needed.

## Conclusion

High Lp(a) levels, found in 20% of the population, increase the risk of cardiovascular disease (CVD) by 2–3-fold, yet there are no current treatments to address this issue. In this case, the patient experienced a 39% reduction in Lp(a) following a 10-day water-only fast and 6 weeks of strict adherence to an SOS-free diet. This reduction is comparable to that achieved with PCSK9 inhibitors, which improve CVD outcomes. Additionally, the patient showed improvements in other CVD risk markers, suggesting that normal-weight people with a vegan diet may still benefit from this intervention. Further research is necessary to determine if prolonged water-only fasting and/or an SOS-free diet can effectively reduce CVD risk in this population.

## Data Availability

The original contributions presented in the study are included in the article/supplementary material, further inquiries can be directed to the corresponding author.
